# Long-term QALY-weights among spouses of dependent and independent midlife stroke survivors

**DOI:** 10.1007/s11136-017-1636-z

**Published:** 2017-06-29

**Authors:** Josefine Persson, Mattias Aronsson, Lukas Holmegaard, Petra Redfors, Kaj Stenlöf, Katarina Jood, Christina Jern, Christian Blomstrand, Gunilla Forsberg-Wärleby, Lars-Åke Levin

**Affiliations:** 1 Department of Clinical Neuroscience, Institute of Neuroscience and Physiology, Sahlgrenska Academy, University of Gothenburg, Gothenburg, Sweden; 2 Health Metrics, Sahlgrenska Academy, University of Gothenburg, Gothenburg, Sweden; 3 Department of Medical and Health Science, Linköping University, Linköping, Sweden; 4 Department of Gastrosurgical Research and Education, Institute of Medicine, Sahlgrenska Academy, University of Gothenburg, Gothenburg, Sweden; 5 Department of Clinical Pathology and Genetics, Institute of Biomedicine, Sahlgrenska Academy, University of Gothenburg, Gothenburg, Sweden

**Keywords:** Stroke, Spouses, Health-related quality of life, Quality adjusted life years, SF-36, SF-6D

## Abstract

**Purpose:**

The aim of this study was to investigate whether the dependency of midlife stroke survivors had any long-term impact on their spouses’ QALY-weights.

**Method:**

Data on stroke survivors, controls, and spouses were collected from the 7-year follow-up of the Sahlgrenska Academy Study on Ischemic Stroke. Health-related quality of life was assessed by the SF-36, and the preference-based health state values were assessed with the SF-6D. Spouses of dependent and independent stroke survivors were categorized according to their scores on the modified Rankin Scale. An ordinary least squares regression analysis was used to evaluate whether the dependency of the stroke survivors had any impact on the spouses’ QALY-weights.

**Result:**

Cohabitant dyads of 247 stroke survivors aged <70 at stroke onset and 245 dyads of controls were included in the study. Spouses of dependent stroke survivors (*n* = 50) reported a significant lower mean QALY-weight of 0.69 in comparison to spouses of independent stroke survivors (*n* = 197) and spouses of controls, (*n* = 245) who both reported a mean QALY-weight of 0.77. The results from the regression analysis showed that higher age of the spouse and dependency of the stroke survivor had a negative association with the spouses’ QALY-weights.

**Conclusion:**

The QALY-weights for spouses of dependent midlife stroke survivors were significantly reduced compared to spouses of independent midlife stroke survivors. This indicates that the inclusion of spouses’ QALYs in evaluations of early treatment and rehabilitation efforts to reduce stroke patients’ dependency would capture more of the total effect in dyads of stroke survivors.

## Introduction

Health economic evaluations are an important basis for resource allocation and priority setting decisions within health care. Quality adjusted life years (QALYs) are the recommended outcome measure for such analyses of new treatment strategies and interventions [1–3]. QALYs combines life years and health-related quality of life (HRQoL) using a single index on a 0–1 scale, where 0 equals death and 1 represents perfect health. This index is often called QALY-weight, and one QALY corresponds to a year of “perfect” health.

Economic evaluations of treatment strategies have an increasing impact on the allocation of the scarce resources in the health care sector [4]. Economic evaluations tend to focus on the health consequences concerning the patients. However, an economic evaluation with a societal perspective should ideally include all costs and effects that arise due to an intervention, thus, also consequences for the relatives, particularly if they are significant [5]. However, this is rarely done due to insufficient empirical data, which in turn may lead to policy decisions with undesirable allocation effects [6] and ultimately poorer population health.

One important group to include in economic evaluations is informal caregivers, usually family members, who provide many hours of unpaid informal support to the patient at home [7]. Spouses of stroke survivors provide support to their partner to an extent that far exceeds what is normally offered by society [8]. To support a family member is often perceived as natural by the spouse, but can at the same time be experienced as a burden and may have a negative impact on their own health [9]. Most studies include relatives of elderly stroke survivors, while there is less knowledge about the HRQoL of relatives of younger stroke survivors. Caregivers to younger stroke survivors are often themselves in midlife with responsibility for family and working life [10]. There are indications in earlier studies with a short-term perspective that both the age of the caregiver and the functional status of the stroke survivors are determinants of reduced HRQoL [11]. In a recent study, we showed that the HRQoL of spouses to stroke survivors is affected by the global disability of the stroke survivors also in a long-term perspective [12]. However, to analyze how this affects the valuation of the spouses’ QALY-weights, we need to apply a preference-based valuation instrument. Further, when evaluating the cost-effectiveness of a stroke intervention with a societal perspective that aims to reduce the dependency of the stroke survivors, it is of importance to know whether the intervention also have an impact on the spouses QALY-weight. Thus, including a possible positive effect on the spouses QALY-weight might have an impact on the cost-per-QALY estimate and thereof the decision-making. The objective of this study was to investigate whether the dependency of the midlife stroke survivors had any long-term impact on their spouses’ QALY-weight values.

## Methods

### Subjects

The participants in this study were from the Sahlgrenska Academy Study on Ischemic Stroke (SAHLSIS), the design of which has been reported previously [12, 13]. In brief, 600 patients with ischemic stroke before the age of 70 were consecutively recruited from four stroke units within western Sweden between 1998 and 2003. The hospitals have identical principles for acute stroke care, basal diagnostic set-up, and stroke unit care. The stroke survivors were age, sex, and geographically matched with 600 controls who were recruited from a population-based health survey or the population registry. For the 7-year follow-up, during 2006–2012, patients and controls were invited to respond to a questionnaire regarding background variables and self-rating instruments concerning health issues. In addition, stroke survivors included at the Sahlgrenska University hospital were invited to visit the research physician and research nurse. Cohabitant spouses of both stroke survivors and controls at the 7-year follow-up were invited to respond to a questionnaire regarding sociodemographic measures and a self-rating instrument for HRQoL. The recruited participants and drop-out rates have been presented in detail elsewhere [12]. In brief, at SAHLSIS baseline, 422 stroke survivors and 437 controls were cohabitant. In the 7-year follow-up, 299 stroke survivors and 344 controls were cohabitant, whereof 248 spouses of stroke survivors and 245 spouses of controls were recruited to the study. In this study, one patient had missing data for mRS, and thus, 247 dyads of stroke survivors were included. All respondents gave informed consent and approved merging of their data for the current study. The studies were approved by the Regional Ethics Committee in Gothenburg (Reference Number 413-04, 622-06).

### Assessments

Sociodemographic data for the study population were collected from the SAHLSIS database [12, 13]. The HRQoL was assessed using the Short Form-36 (SF-36) questionnaire (version 1) in an approved Swedish version [14]. The SF-36 consists of eight scales: physical functioning, physical role, bodily pain, general health, vitality, social functioning, emotional role, and mental health. The first four are physical scales and latter four are mental scales. To derive a preference-based measure of health from the SF-36, an algorithm developed by Brazier et al. [15] was used to revise the SF-36 questionnaire into the six-dimensional health state classification, SF-6D. The SF-6D consists of the following dimensions: physical functioning, role participation (combined role-physical and role-emotional), social functioning, bodily pain, mental health, and vitality. Each dimension has between four and six response levels, resulting in 18,000 different health states, which have been weighted directly or indirectly using the standard gamble method on a random sample of the general population in the United Kingdom.

For the stroke survivors, the modified Rankin Scale (mRS) [16] was used as a measure of global disability that also affects dependency. The scale is defined categorically with seven different grades: 0 indicates no symptoms at all, 1 indicates no significant disability despite symptoms (able to carry out all usual duties and activities), 2 indicates slight disability (unable to carry out all previous activities, but able to look after own affairs without assistance), 3 indicates moderate disability (requiring some help, but able to walk without assistance), 4 indicates moderately severe disability (unable to walk without assistance and unable to attend to own bodily needs without assistance), 5 indicates severe disability (bedridden, incontinent, and requiring constant nursing care and attention) and 6 indicates death. A score of 0–2 indicates independence, while a score of 3–5 indicates dependence [17]. Outcome was also assessed with the National Institutes of Health Stroke Score (NIHSS) [18], which provides a quantitative measure of stroke-related neurological impairment. A higher score indicates a more severe outcome. The ability to perform basic activities of daily living was assessed by the barthel index (BI) [19], with a score between 0 and 100, where a higher score indicates better abilities in self-care. Cognitive impairment was assessed by the Barrow Neurological Institute Screen for Higher Cerebral Functions (BNIS) [20, 21]. A lower score reflects lower cognitive function. Anxiety and depression were self-assessed by the Hospital Anxiety and Depression Scale (HADS-D for depression and HADS-A for anxiety) [22], and a high score reflects presence of anxiety/depressive symptoms. The BNIS was scored by the research nurse for patients who came to the Sahlgrenska University Hospital for the 7-year follow-up.

### Statistical analyses

The distribution of the variables was presented as mean and SD or median and first (Q1) and third (Q3) quartile for continuous variables and as number and percentage for categorical variables. All significance tests were two-sided and conducted at the 5% significance level. For comparison between spouses of dependent and independent stroke survivors as well as dependent and independent stroke survivors, Mann–Whitney *U* test was used due to skewed data. However, SF-6D data were normally distributed and hence a *t* test was used for comparison between spouses of dependent and independent stroke survivors. To investigate the relationship between the spouses’ QALY-weights and the stroke survivors’ global disability, we used ordinary least squares regression with the health state value as the outcome variable and the spouses’ demographic features and stroke-related variables as explanatory variables. Due to high co-linearity between the mRS, NIHSS, and Barthel Index, only the primary explanatory variable mRS was included in the model to interpret the effect. The other explanatory variables in the models were the spouses’ age, sex, and educational level and the stroke survivors’ BNIS, HADS-D, and HADS-A. To investigate whether the dependency of the stroke survivors had any impact on their spouses’ QALY-weights, in model 1, mRS was included as dichotomized scores of dependency (mRS score 3–5) and independency (mRS score 0–2, set as a reference). To investigate which of the mRS scores had the highest impact on the spouses’ QALY-weights, in model 2, mRS was included as a categorical variable, where mRS score 0 was set as a reference. Due to few responders having mRS score 5, mRS scores 4 and 5 were merged into one category. Spouses’ age was categorized as <50, 50–65, and >65, where <50 was set as the reference. The model was constructed using a purposeful stepwise regression approach. A corresponding method but for logistic regressions has been described by Bursac et al. [23]. In the first step, the ANOVA was tested with one variable at a time to examine whether the *F* test had a *P* ≤ 0.25. If so, the variable was included in the large model (Age reference (<50) vs 50–65 *P* = 0.520 and vs >65 *P* = 0.016, Sex *P* = 0.851, Education *P* = 0.939, mRS reference (0) vs 1 *P* = 1, vs 2 *P* = 0.938, vs 3 *P* = 0.003, vs 4 *P* = 0.175, mRS reference (independence) vs dependence *P* < 0.001, BNIS *P* < 0.001, HADS-D *P* = 0.009, HADS-A *P* < 0.283). In the second step, the variables with *P* > 0.10 were removed from the large model, and only variables with *P* < 0.10 were included in the final model (Age reference (<50) vs 50–65 *P* = 0.322 and vs >65 *P* = 0.024, mRS reference (0) vs 1 *P* = 0.993, vs 2 *P* = 0.462, vs 3 *P* = 0.002, vs 4 *P* = 0.087, mRS reference (independence) vs dependence *P* < 0.001, BNIS *P* = 0.517, HADS-D *P* = 0.811). In the final step, the variables not included in the final model were reintroduced to the final model with one variable at a time to examine whether the *F* test had a *P* ≤ 0.25, although none of the variables met this criteria (BNIS *P* = 0.377, HADS-D *P* = 0.261, HADS-A *P* = 0.684, Spouses sex *P* = 0.730 and Spouses education *P* = 0.762). All the analyses were carried out in SPSS software (version 23, SPSS, Inc., Chicago, IL, USA).

## Results

The population of this study consisted of 247 cohabitant dyads of stroke survivors and 245 cohabitant dyads of healthy controls. The mean (SD) ages of the spouse and the stroke survivors were 63 (11) and 64 (11), respectively, and 66 and 34% were females, respectively. As assessed by the mRS data at the 7-year follow-up, 50 were spouses of dependent (mRS score 3–5) stroke survivors, and 197 were spouses of independent (mRS score 0–2) stroke survivors. Among the dependent stroke survivors, 20 received any form of formal support, whereof 80% had a mRS score of 4 or 5. The demographic features of the study population are shown in Table 1.

**Table 1:** Demographic features of the sample

	Spouses of dependent stroke survivors (%) (*n* = 50)	Spouses of independent stroke survivors (%) (*n* = 197)	Spouses of controls (%) *n* = 245	Dependent stroke survivors (%) (*n* = 50)	Independent stroke survivors (%) (*n* = 197)	Controls (%) (*n* = 245)
Mean age, years (SD)	67 (8)	62 (11)	64 (9)	68 (8)	63 (11)	65 (9)
Female sex	31 (63)	131 (67)	161 (66)	19 (38)	65 (33)	84 (34)
Education						
Secondary or less	24 (48)	72 (37)	71 (29)	21 (42)	70 (36)	83 (34)
High school	11 (22)	65 (33)	89 (36)	17 (34)	70 (36)	87 (36)
University	15 (30)	60 (30)	85 (35)	11 (22)	57 (28)	74 (30)
Occupation^a^						
Employed	13 (26)	92 (47)	97 (38)	0 (0)	64 (32)	103 (40)
Retired	35 (70)	91 (46)	132 (52)	38 (76)	110 (56)	145 (56)
Unemployed, sick leave, other	7 (14)	25 (13)	26 (10)	13 (26)	57 (29)	12 (5)
Household						
Children <18 at Support in home	2 (4)	25 (13)	22 (9)			
Informal support^b^	48 (96)	31 (16)	8 (3)			
Formal support^c^				20 (40)	4 (0.02)	(0)
mRS score						
0					39 (20)	
1					53 (27)	
2					105 (53)	
3				25 (50)		
4–5				25 (50)		

^a^Sum not equal to 100% because of multiple response alternatives

^b^Self-reported information from the spouse concerning whether they provided informal support to their partner

^c^Home care, personal assistant, or living at nursing home

**Fig. 1:**
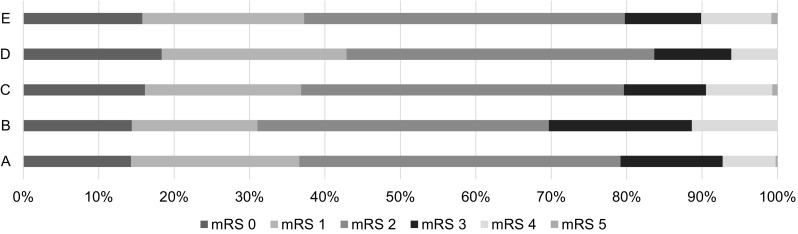
mRS scores for stroke survivors included in the study compared to those not eligible for inclusion (“non eligible”) in this study from the SAHLSIS seven-year follow-up. **a** mRS scores at 3 months for cohabitant stroke survivors (*n* = 422, missing mRS data for 19 stroke survivors), **b** mRS scores at 3 months for “non eligible” stroke survivors before the seven-year follow-up (*n* = 131), **c** mRS scores at 7 years for cohabitant stroke survivors (*n* = 299; missing mRS data for 14 stroke survivors), **d** mRS scores at 7-years for excluded stroke survivors because researchers were not permitted to contact the spouse or because the spouse declined participation (*n* = 51; missing mRS data for 1 stroke survivors), **e** mRS scores at 7 years for included stroke survivors (*n* = 247)

The stroke survivors who were lost between baseline and the seven-year follow-up had worse global disability, measured with the mRS (*P* < 0.001) (Fig. 1). No age differences were seen between the stroke survivors who were lost before the seven-year follow-up and those included in this study; however, more males than females were lost to follow-up. Of the cohabitant stroke survivors at baseline (*n* = 422), 21% were dependent stroke survivors according to their mRS score. Among the stroke survivors lost before the 7-year follow-up (*n* = 131), 31% were dependent stroke survivors. Further, of the stroke survivors who were cohabitant at the seven-year follow-up (*n* = 299) and those included in the study (*n* = 247), 20% were dependent stroke survivors. Of the cohabitant stroke survivors at the 7-year follow-up (*n* = 299) who did not give permission to contact their spouse or their spouse decline participation, 16% were dependent stroke survivors. Of the included stroke survivors (*n* = 247), 16.5% had a recurrent stroke with increased dependency since baseline. In this group of stroke survivors with recurrent stroke (*n* = 41), 16% were dependent at baseline while 38% were dependent at 7 year.

The dependent stroke survivors had more impaired neurological and cognitive function, higher level of depressive symptoms, and more impaired activities of daily living compared to the independent stroke survivors (Table 2). Data concerning the cognitive function (BNIS) and neurological impairment (NIHSS) were available for a subgroup at the Sahlgrenska University hospital. There were no statistical significant differences for the other stroke-related variables (mRS, Barthel Index, HADS-A and HADS-D) for stroke survivors from the Sahlgrenska University hospital (*n* = 176) in comparison with stroke survivors from the other three stroke units (*n* = 71). At the 7-year follow-up, 30% of the dependent stroke survivors and 13% of the independent stroke survivors had had a recurrent stroke.

**Table 2:** Stroke-related measures for dependent and independent stroke survivors

	Dependent stroke survivors (mRS ≥ 3) (*n* = 50)	Independent stroke survivors (mRS ≤ 2) (*n* = 197)	*P* value*
	Median (Q_1_–Q_3_)	Median (Q_1_–Q_3_)	
Neurological impairment (NIHSS)^a^	6 (2–12)	0 (0–0)	<0.001
Cognitive function (BNIS)^a^	32 (27–37)	41 (38–45)	<0.001
Depression (HADS-D)^b^	6 (2–11)	3 (1–6)	<0.001
Anxiety (HADS-A)^b^	4 (1–8)	3 (1–6)	0.145
Barthel Index^b^	75 (49–90)	100 (100–100)	<0.001

^a^Subgroup from the Sahlgrenska University hospital (*n* = 170)

^b^Total study population (*n* = 247)

* Mann–Whitney *U* Test

Spouses of dependent stroke survivors reported significantly lower HRQoL in all SF-36 scales compared to spouses of independent stroke survivors and spouses of controls (Table 3). Spouses of dependent stroke survivors reported a mean (SD) QALY-weight of 0.69 (0.12) in comparison to both spouses of independent stroke survivors and spouses of controls, each group having a mean QALY-weight of 0.77 (0.11). The difference in mean QALY-weight between spouses of dependent and independent stroke survivors (0.08 points) was statistically significant (*P* < 0.001). However, there was no statistical difference in mean QALY-weights between female and male spouses.

**Table 3:** Health-related quality of life domains for spouses of independent and dependent stroke survivors and spouses of controls. *Source* Domains are from the SF-36 scale (10)

	Spouses of dependent stroke survivors (*n* = 50)	Spouses of independent stroke survivors (*n* = 197)	Spouses of controls (*n* = 245)^a^	*P* value^b^
	Mean (SD) Median (Q_1_–Q_3_)	Mean (SD) Median (Q_1_–Q_3_)	Mean (SD) Median (Q_1_–Q_3_)	
Physical functioning	71 (27) 78 (50–95)	87 (17) 95 (80–100)	87 (18) 95 (80–100)	<0.001
Physical role	66 (37) 75 (25–100)	82 (35) 100 (75–100)	86 (32) 100 (100–100)	<0.001
Bodily pain	65 (29) 62 (41–100)	74 (26) 84 (51–100)	76 (26) 84 (52–100)	0.047
General health	57 (23) 52 (37–77)	76 (21) 78 (62–92)	77 (21) 82 (66–95)	<0.001
Vitality	53 (27) 53 (30–76)	70 (22) 75 (60–85)	73 (22) 80 (60–90)	<0.001
Social functioning	76 (23) 75 (63–100)	90 (20) 100 (88–100)	97 (19) 100 (88–100)	<0.001
Emotional role	65 (39) 67 (33–100)	90 (32) 100 (100–100)	90 (27) 100 (100–100)	<0.001
Mental health	67 (22) 70 (52–84)	81 (18) 88 (76–92)	84 (16) 88 (76–96)	<0.001

^a^Published in Persson et al. [12]

^b^Mann–Whitney *U* Test for spouses of dependent vs independent stroke survivors

**Fig. 2:**
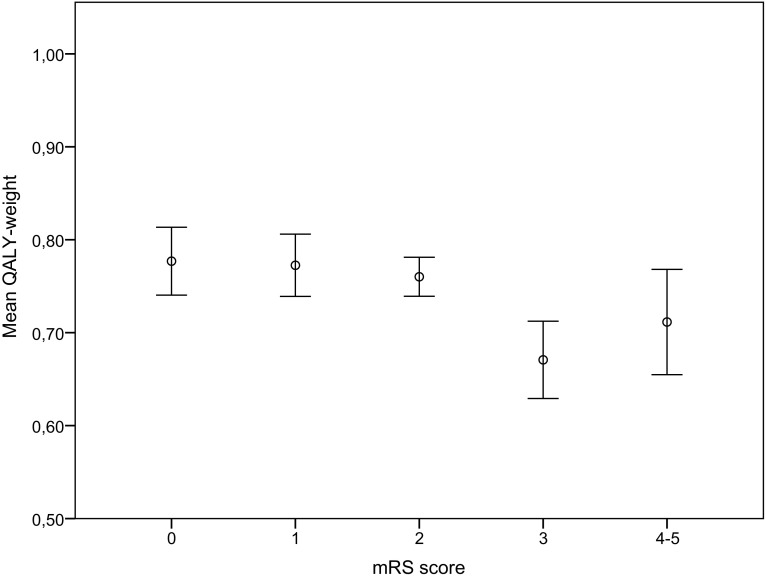
Spouses’ mean QALY-weights in each of the stroke survivors mRS score, including 95% CI error bars

The results from the ordinary least squares regression analyses showed that higher age of the spouse and higher mRS score of the stroke survivors were associated with lower QALY-weights of the spouses of stroke survivors (Table 4). Model 1 showed that the dependency of the stroke survivor had a significantly negative association with the spouses’ QALY-weights. Model 2 showed that mRS score of 3, compared to 0, had a significantly negative association with the spouses’ QALY-weights, also illustrated by Fig. 2.

**Table 4:** Regression analysis concerning QALY-weights for spouses of stroke survivors

	Model 1		Model 2	
	Coefficient	*P* value	Coefficient	*P* value
Spouses’ age				
50–65^a^	−0.025	0.299	−0.024	0.322
>65^a^	−0.054	0.025	−0.055	0.024
Stroke survivors’ mRS score				
Dependency (mRS score 3–5)^b^	−0.065	<0.001		
mRS score 1^c^			0.000	0.993
mRS score 2^c^			−0.016	0.462
mRS score 3^c^			−0.094	0.002
mRS score 4–5^c^			−0.051	0.087
Constant	0.802	<0.001	0.827	<0.001
Observations	244		244	
*R* ^2^	0.092		0.103	

The ordinary least square regression analyses are based on a sample of 244 due to missing SF-6D index for 3 spouses

Reference categories

^a^Age < 50

^b^Independency (mRS score 1–2)

^c^mRS score 0

## Discussion

In this long-term follow-up, we found that spouses of dependent midlife stroke survivors reported lower QALY-weights compared with spouses of independent midlife stroke survivors and spouses of healthy controls with a difference in mean larger than 0.03 points, i.e., the recommended minimally important difference for evaluative purposes [24]. To the author’s knowledge, this is the first study estimating QALY-weights for spouses of stroke survivors that used the SF-6D. According to a systematic literature review of caregivers for patients with various diseases [25], knowledge concerning the spill-over effect of QALY-weight decrements of illness on family members and caregivers is limited. The review, however, indicates that the spill-over effects measured with QALY-weights are generally small. A recent study by Gupta et al. [26] showed a mean (SD) QALY-weight measured by SF-6D of 0.64 (0.12) for schizophrenia caregivers compared to 0.67 (0.13) for caregivers for adult relatives with other conditions, such as dementia and stroke. Thus, this study with caregivers to adults with various illnesses indicated QALY-weights comparable to the spouses of dependent stroke survivors in our study. Subsequently, a study by Ågren et al. [27] showed a mean (SD) QALY-weight, measured with SF-36 converted into a mean EQ-5D preference-based score, of 0.79 (0.21) for caregivers to patients with chronic heart failure. The caregivers QALY-weights indicated no significant difference compared to the reference group in the normal population. These results are comparable to the spouses of independent stroke survivors and spouses of controls in our study. A study by Patel et al. [28] evaluating a training program for caregivers of stroke survivors showed that at baseline, the mean (SD) QALY-weights for trained and untrained caregivers were 0.94 (0.10) and 0.94 (0.14), respectively, when measured by EQ-5D, with no significant difference between the two groups. This result is in line with an intervention study of befriending services for carers of people with dementia [29], showing a mean QALY-weight of 0.95 in the intervention group and 0.93 in the control group when measured by EQ-5D, with no significant difference between the two groups. The results of these two studies indicate higher QALY-weights measured with EQ-5D compared to our QALY-weight results measured with the SF-6D. It has previously been shown that there are differences in scores between SF-6D and EQ-5D, where SF-6D focuses more on the social functioning and the EQ-5D gives more weight to the physical functioning [30], and comparisons between these different measures should be taken with caution. Thus, the SF-6D provides a broader view of the individuals’ health and participation in life. This might be especially appropriate for capturing the impact on QALY-weights among individuals who do not experience any physical impairment, i.e., caregivers [7] and also the general population. This might also be the reason why the QALY-weights of the spouses of controls in our study were somewhat lower compared to the health index measured by the EQ-5D for the Swedish normal population [31].

The regression analysis in our study showed that the mRS score of the stroke survivors was an explanatory variable for lower QALY-weights of spouses of stroke survivors, which was also found in previous studies [11]. The regression models also showed that a mRS score of 3 had a significant negative association with the spouses’ QALY-weights, thus indicating that spouses of dependent stroke survivors report lower QALY-weights compared to spouses of independent stroke survivors. It is interesting to note that the regression model showed that a mRS score of 4–5 did not have a significant association with the spouses’ lower QALY-weights. This might be due to different reasons, whereof one possible explanation might be that these dyads have access to formal care to a greater extent compared to spouses for stroke survivors with mRS score 3. However, this study population was too small to investigate the discrepancy in QALY-weights, and further studies are necessary.

To the authors’ knowledge, this is the first study estimating QALY-weights for spouses of independent and dependent stroke survivors. A Swedish study [32] of caregivers with a supportive role indicated that the more extensive the support towards the family member with a disease/condition, the more impact it has on the caregivers’ HRQoL. Furthermore, a study from the Netherlands reported an association between the experienced burden of primary caregivers of stroke patients and lower QALY-weights measured with EQ-5D [33]. According to a systematic review of the use of EQ-5D with caregivers of patients with dementia, the severity of the dementia did not have a negative impact on the caregivers’ self-reported health, although the perceived burden and time spent on caring was strongly related to the caregivers’ self-reported health [34]. Thus, even when comparing across diseases, perceived burden and provided informal support have a negative impact on the caregivers’ own health.

Methods for estimations of health spill-over effects on the family and how to include that effect in economic evaluations have been frequently discussed in the literature [35, 36]. However, the methods used in our study were not intended to capture the direct spill-over effects of the stroke disease. The aim of our study was rather to capture the spill-over effects related to the dependency of the stroke survivor. Since the dependency of the stroke survivor is highly associated with support from both the society and the family, it would be difficult to disentangle the spill-over effects of the dependency from the provided formal and informal support. Hence, the aim was instead to investigate whether the dependency of stroke survivors itself had any impact on their spouses’ QALY-weight. The rationale for this was that in our previous study, we found that spouses of stroke survivors report reduced HRQoL in comparison to spouses of controls [12]. However, the results of the current study show that there is a significant difference in the spouses’ HRQoL, depending upon whether the stroke survivors are scored as independent or dependent, defined by the mRS. This is of importance, both in economic evaluations of new health care treatments with a societal perspective, where mRS is often used as the most important driver in models of stroke treatments, and in decision making by clinicians and rehabilitation stakeholders with regard to targeted interventions for families of stroke survivors.

The advantage of studying this well-documented population with consecutively included stroke survivors is that it highlights a population in the middle of life with responsibility for family and working life, 7 years after stroke onset, whereas most studies have focused on the older population in a shorter time-perspective. The study has some limitations. Of the dependent stroke survivors, 10% stated that their spouse or the research nurse or physician completed the instruments that should have been self-assessed, i.e., the HADS-A and HADS-D. This might have influenced the results for these stroke survivors. Another limitation of this study is that we solely had longitudinal data for the stroke survivors and the controls, and not for the spouses. Therefore, it was not possible to include spouses to patients who died or spouses who divorced during follow-up. Furthermore, a larger proportion of stroke survivors with higher mRS scores at 3 months were lost to follow-up at 7 years [12], probably underestimating the QALY-weights of the spouses of dependent stroke survivors in this study. Further longitudinal studies are needed to investigate the impact of stroke disease on spouses’ health.

In conclusion, the QALY-weights for spouses of dependent midlife stroke survivors were significantly decreased compared to spouses of independent midlife stroke survivors. This indicates that reduced dependency through early treatments and effective rehabilitation might have the potential to increase the spouses’ QALY-weights to levels comparable to spouses of healthy controls. The inclusion of spouses’ QALYs in economic evaluations of treatments for stroke patients would capture more of the total effect in dyads of stroke survivors.
